# Frailty and survival of patients with renal cell carcinoma: A meta-analysis

**DOI:** 10.17305/bb.2025.12687

**Published:** 2025-07-18

**Authors:** Longye Zhang, Weiping Liu, Bo Ning, Bohan Chen

**Affiliations:** 1 Department of Nephrology, The First Hospital of Qinhuangdao, Qinhuangdao, China; 2 Department of Urology, Huludao Central Hospital, Huludao, China

**Keywords:** Renal cell carcinoma, RCC, frailty, survival, progression, meta-analysis

## Abstract

Frailty is a multidimensional syndrome reflecting decreased physiological reserve and increased vulnerability to stressors, which may adversely affect cancer prognosis. However, its impact on survival outcomes in patients with renal cell carcinoma (RCC) remains unclear. This meta-analysis aimed to evaluate the association between frailty and survival in RCC patients. A systematic search of PubMed, Embase, and Web of Science was conducted for longitudinal studies assessing frailty in adults with RCC. Studies using validated frailty assessment tools and reporting overall survival (OS) and/or progression-free survival (PFS) were included. Pooled hazard ratios (HRs) and 95% confidence intervals (CIs) were calculated using random-effects models. Subgroup and sensitivity analyses were performed to explore heterogeneity. Eight cohort studies involving 15,989 RCC patients were included. Frailty was associated with significantly poorer OS (HR ═ 1.79, 95% CI: 1.45–2.20; *I*^2^ ═ 30%) and PFS (HR ═ 2.17, 95% CI: 1.54–3.04; *I*^2^ ═ 0%). The association between frailty and OS remained robust across sensitivity analyses by excluding one study at a time and was consistent across subgroups stratified by cancer stage, treatment modality, patient age, frailty assessment method, follow-up duration, and analytic model (all *P* values for subgroup differences >0.05). Subtype-specific data according to the histologic type of RCC were unavailable, which limits detailed prognostic interpretation. No significant publication bias was detected. Frailty may be significantly associated with poorer survival outcomes in patients with RCC. Incorporating frailty assessment into routine clinical evaluation may aid in prognostication and individualized treatment planning for this patient population.

## Introduction

Renal cell carcinoma (RCC) is the most common type of kidney cancer, accounting for approximately 90% of all renal malignancies [1, 2]. Globally, RCC ranks as the sixth and tenth most common cancer among men and women, respectively, with incidence rates steadily increasing [3]. While early-stage RCC may be effectively treated with surgical resection, a significant proportion of patients present with advanced or metastatic disease at diagnosis or experience recurrence following initial therapy [4, 5]. The prognosis of RCC varies considerably depending on stage, histologic subtype, and patient characteristics, with 5-year survival rates ranging from over 90% for localized disease to less than 15% for metastatic cases [6–8]. Advances in targeted therapies and immune checkpoint inhibitors have improved outcomes in recent years; however, survival remains suboptimal in high-risk patients [9, 10]. As such, identifying robust predictors of poor survival is crucial for optimizing treatment decisions, individualizing care, and improving clinical outcomes in patients with RCC [11].

Frailty is a multidimensional syndrome characterized by reduced physiological reserve and impaired response to stressors, commonly observed in older adults [12]. It reflects the cumulative burden of aging-related deficits across multiple domains, including physical performance, nutritional status, cognition, and comorbidities [13, 14]. In oncology, frailty has gained increasing attention as a clinically relevant prognostic indicator, influencing treatment tolerance, recovery, and survival [15, 16]. In patients with cancer, including RCC, frailty may contribute to poor prognosis through mechanisms such as impaired immune surveillance, delayed recovery from therapy, and increased susceptibility to complications [15, 16]. Although individual studies have suggested an association between frailty and survival outcomes in RCC, the findings remain inconsistent, and no meta-analysis has comprehensively synthesized the available evidence [17, 18]. Given the growing clinical emphasis on precision oncology and risk stratification, understanding the prognostic role of frailty in RCC could provide valuable insights for pre-treatment assessment and therapeutic planning [11]. Therefore, this meta-analysis aimed to evaluate the association between frailty and survival outcomes—including overall survival (OS) and progression-free survival (PFS)—in patients with RCC.

## Materials and methods

This meta-analysis was conducted in accordance with the PRISMA 2020 statement [19, 20] and the Cochrane Handbook for Systematic Reviews [21], which guided the development of the protocol, data collection, statistical synthesis, and reporting. The protocol has been prospectively registered in the PROSPERO database under the identifier CRD420251056657 (https://www.crd.york.ac.uk/PROSPERO/view/CRD420251056657).

### Database search

To retrieve studies according to the aim of this meta-analysis, we searched the PubMed, Embase, and Web of Science databases using an extensive array of search terms, which included: (1) “frailty” OR “frail”; (2) “renal” OR “kidney”; (3) “cancer” OR “tumor” OR “carcinoma” OR “neoplasm” OR “adenoma” OR “malignancy”; and (4) “recurrence” OR “death” OR “mortality” OR “survival” OR “prognosis” OR “deaths” OR “remission” OR “collapse” OR “follow-up” OR “followed” OR “metastasis” OR “progression” OR “longitudinal” OR “cohort” OR “died”. The literature search was limited to studies involving human participants and included only full-length, peer-reviewed articles published in English. To ensure comprehensive coverage, the reference lists of relevant original and review articles were also manually screened for additional eligible studies. The search spanned from the inception of each database through April 10, 2025, with the full search strategies detailed in Supplemental data.

### Study selection

The inclusion criteria were structured according to the PICOS framework.

Population (P): Adults aged 18 years or older with confirmed a diagnosis of RCC, regardless of cancer stage and main anticancer treatment.

Exposure (I): Patients with frailty, which was diagnosed according to the methods and scales in the original studies.

Comparison (C): Patients without frailty.

Outcome (O): Survival outcomes, including OS and PFS, compared between patients with and without frailty. In general, OS is defined as the time from treatment initiation to death from any cause, while PFS is defined as the time from treatment initiation to disease progression or death, whichever occurs first.

Study design (S): Longitudinal observational studies, including cohort studies, nested case-control designs, and post-hoc analyses of clinical trials.

Exclusion criteria included reviews, editorials, meta-analyses, preclinical studies, and studies that included participants with other cancers, lacked a defined measure of frailty, or did not report the survival outcomes. In cases of overlapping populations, the study with the largest and most complete dataset was included.

### Study quality evaluation and data collection

The literature search, study selection, quality assessment, and data extraction were conducted independently by two reviewers, with any disagreements resolved through discussion with the corresponding author. Study quality was evaluated using the Newcastle–Ottawa Scale (NOS), which assesses three domains: participant selection, control for confounding, and outcome assessment [22]. The NOS assigns scores from 1 to 9, with higher scores indicating better quality; studies scoring 7 or above were classified as high quality. Extracted data included study-level information (first author, publication year, country, and study design), participant characteristics (diagnosis, main anticancer treatment, number of subjects, mean age, sex distribution, and cancer stage), details on the scales used to evaluate frailty and the number of patients with frailty, median follow-up durations, survival outcomes reported, and the covariates adjusted for in the association analyses.

### Statistical analysis

The association between frailty and OS/PFS in patients with RCC was evaluated by pooling hazard ratios (HRs) and their corresponding 95% confidence intervals (CIs), comparing individuals with and without frailty. When necessary, HRs and their standard errors were calculated from reported 95% CIs or *P* values and then log-transformed to stabilize variance and normalize the distribution [21]. Between-study heterogeneity was assessed using the Cochrane *Q* test and the *I*^2^ statistic, with thresholds of <25%, 25%–75%, and >75% interpreted as low, moderate, and high heterogeneity, respectively [23]. A random-effects model was applied to account for expected variation across studies [21]. Sensitivity analysis was conducted by sequentially omitting each study to examine the stability of the pooled estimate. Subgroup analyses were also performed to explore the influence of study-level characteristics, such as cancer stage (non-metastatic vs metastatic), main treatment (surgical vs non-surgical), mean ages of the patients (< 65 years vs. ≥ 65 years), methods for evaluating frailty, mean follow-up durations, and analytic model for the association analyses (univariate vs multivariate). Median values of continuous variables were used to define subgroup cutoffs. Publication bias was evaluated through visual inspection of funnel plots and formally tested using Egger’s regression test [6]. A *P* value < 0.05 indicates statistical significance. All statistical analyses were performed using RevMan (version 5.1; Cochrane Collaboration, Oxford, UK) and Stata (version 12.0; Stata Corporation, College Station, TX, USA).

## Results

### Study retrieval

**Figure 1.**
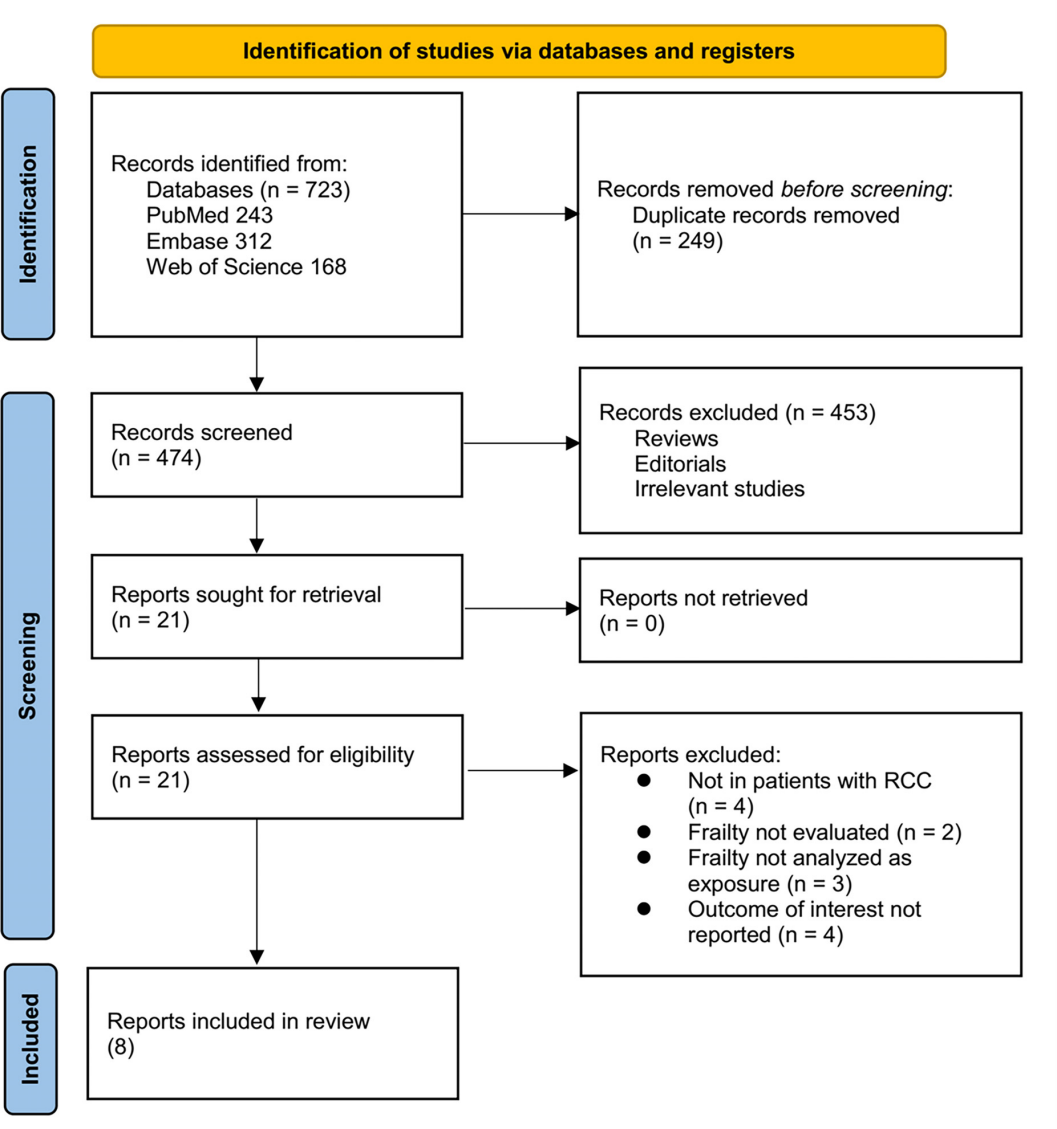
**Flow diagram of study selection.** RCC: Renal cell carcinoma.

The study selection process is illustrated in Figure 1. An initial total of 723 potentially relevant records was identified through database searches and citation screening. After removing 249 duplicates, 474 records remained for title and abstract screening, which resulted in the exclusion of 453 articles that did not align with the meta-analysis objectives. The full texts of the remaining 21 articles were then independently assessed by two reviewers, leading to the exclusion of 13 studies for reasons outlined in Figure 1. Ultimately, eight studies met the inclusion criteria and were included in the quantitative synthesis [24–31].

### Overview of the study characteristics

Table 1 summarizes the characteristics of the eight studies included in this meta-analysis [24–31], published between 2013 and 2023 and conducted in Italy, the United States, China, and Ukraine. All studies were longitudinal cohort designs—seven retrospective [24–26, 28–31] and one prospective [27]—encompassing a total of 15,989 patients with RCC. Study populations varied in mean age from 60.8–77.2 years, with the proportion of male participants ranging from 53.4% to 73.9%. RCC cases included both metastatic and non-metastatic disease (stages I–IV), and patients received treatments such as nephrectomy, systemic therapy, or ablative procedures. Frailty was assessed using various validated tools, including the modified frailty index (mFI) [25, 26, 28, 31], comprehensive geriatric assessment (CGA) [24, 29], Rockwood’s Clinical Frailty Scale (RCFS) [27], and claims-based algorithms [30]. The number of frail patients in each study ranged from 7 to 581, with a total number of 1,117 (7.0%). The median follow-up durations spanned from 1 to 66 months. The outcome of OS was reported in eight studies [24–31], while the outcome of PFS was reported in three [26, 27, 29]. Results of univariate analysis were reported in two studies [24, 27], while data from multivariate analysis were reported in the other six studies [25, 26, 28–31]. Demographic and clinical covariates such as age, sex, tumor size, and stage, etc., were adjusted in multivariate studies. As shown in Table 2, the quality of the included studies was generally moderate to high based on the NOS, with total scores ranging from 6 to 9.

**Table 1:** Characteristics of the included studies

**Study**	**Country**	**Design**	**Diagnosis**	**Main treatment**	**No. of patients**	**Mean age (years)**	**Male (%)**	**Cancer stage**	**Methods for evaluating frailty**	**Number of patients with frailty**	**Median follow-up duration (months)**	**Outcomes**	**Variables adjusted**
Brunello, 2013	Italy	RC	mRCC	Sunitinib	68	74	NR	IV	CGA, based on Balducci’s criteria	7	27.1	OS	None
Lascano, 2015	USA	RC	Non-metastatic RCC	Nephrectomy (partial or radical)	13500	60.8	61	I-III	mFI	581	1	OS	Age, sex, race, smoking status, procedure type
Zhang, 2018	China	RC	Non-metastatic RCC	Nephrectomy (partial or radical)	672	61.7	62.9	I-III	mFI	130	59.6	OS and PFS	Age, sex, BMI, ASA grade, tumor size, pathological T stage, Fuhrman grade
Lesnyak, 2020	Ukraine	PC	T1aN0M0 RCC, tumor size ≤4.0 cm	Radiofrequency ablation or tumor enucleoresection	86	77.2	53.4	I	RCFS	39	60	OS and PFS	None
Pierantoni, 2021	Italy	RC	mRCC	First-line Sunitinib or Pazopanib	86	74.5	64	IV	CGA, based on Balducci’s criteria	15	50	OS and PFS	Age, sex, IMDC risk score, type of TKI
Massaad, 2021	USA	RC	RCC with spinal metastases	Surgery ± PST	88	60.8	73.9	IV	mFI	22	17	OS	Age, sex, ECOG status, IMDC risk group, visceral metastases, sarcopenia, PNI, PST
Spees, 2022	USA	RC	mRCC	Sorafenib, sunitinib, pazopanib, everolimus, axitinib	207	67	70	IV	Claims-based Faurot algorithm	103	24	OS	Age, sex, race, insurance, cancer histology, metastatic diagnosis type, nephrectomy, polypharmacy, health care utilization, provider factors
Rosiello, 2023	Italy	RC	cT1N0M0 RCC	Partial nephrectomy	1282	66.8	73.2	I	mFI	220	66	OS	Age, sex, tumor size, and grade

NR: Not reported; RC: Retrospective cohort; PC: Prospective cohort; mRCC: Metastatic renal cell carcinoma; RCC: Renal cell carcinoma; CGA: Comprehensive geriatric assessment; mFI: Modified frailty index; RCFS: Rockwood’s Clinical Frailty Scale Score; TKI: Tyrosine kinase inhibitor; ASA: American Society of Anesthesiologists; PFS: Progression-free survival; OS: Overall survival; PST: Postoperative systemic therapy; ECOG: Eastern Cooperative Oncology Group; IMDC: International Metastatic RCC Database Consortium; PNI: Prognostic nutritional index.

**Table 2:** Study quality evaluation via the Newcastle–Ottawa scale

**Study**	**Representativeness of the exposed cohort**	**Selection of the non-exposed cohort**	**Ascertainment of exposure**	**Outcome not present at baseline**	**Control for age and sex**	**Control for other confounding factors**	**Assessment of outcome**	**Enough long follow-up duration**	**Adequacy of follow-up of cohorts**	**Total**
Brunello, 2013	0	1	1	1	0	0	1	1	1	6
Lascano, 2015	0	1	1	1	1	1	1	0	1	7
Zhang, 2018	1	1	1	1	1	1	1	1	1	9
Lesnyak, 2020	0	1	1	1	0	0	1	1	1	6
Pierantoni, 2021	0	1	1	1	1	1	1	1	1	8
Massaad, 2021	0	1	1	1	1	1	1	1	1	8
Spees, 2022	0	1	1	1	1	1	1	1	1	8
Rosiello, 2023	0	1	1	1	1	1	1	1	1	8

### Association between frailty and survival of patients with RCC

**Figure 2.**
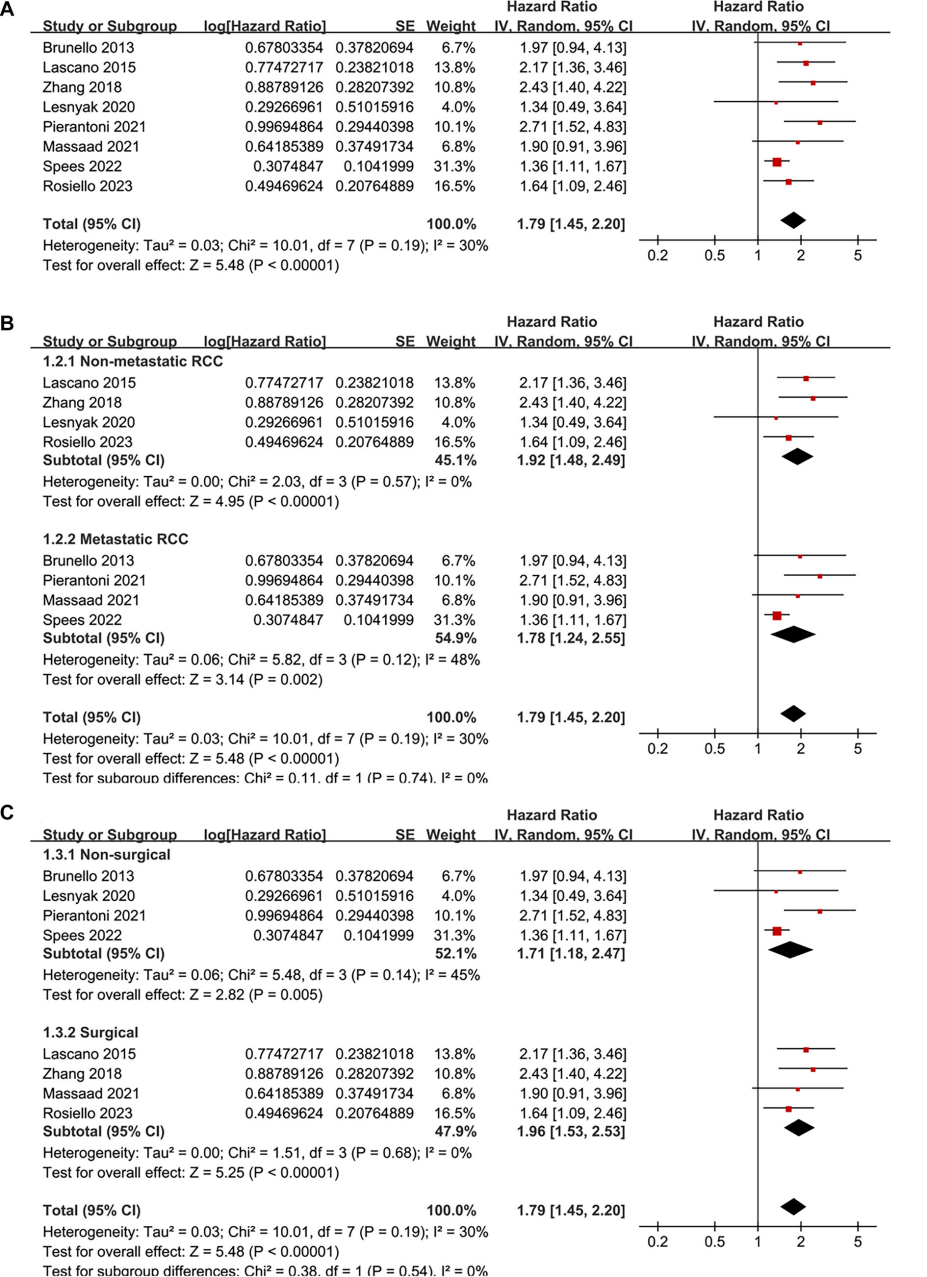
**Forest plot of the association between frailty and OS in patients with RCC.** (A) Pooled analysis comparing patients with and without frailty shows that frailty is significantly associated with poor OS; (B) Subgroup analysis by cancer stage (non-metastatic vs metastatic); (C) Subgroup analysis by type of anticancer treatments (non-surgical vs surgical). OS: Overall survival; RCC: Renal cell carcinoma.

**Figure 3.**
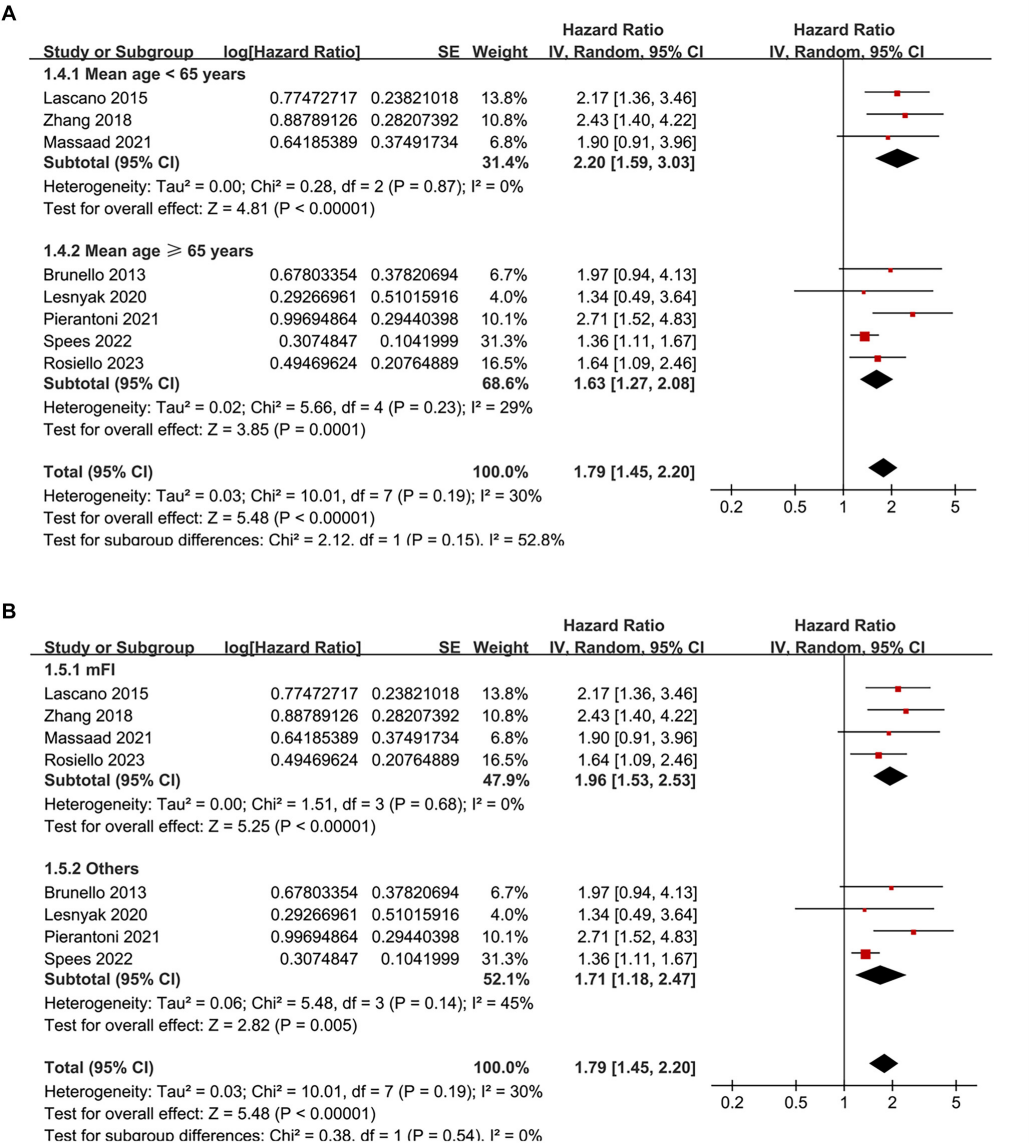
**Subgroup analyses of the association between frailty and OS of patients with RCC.** (A) Stratified by the mean age of the study population (< 65 years vs ≥ 65 years); (B) Stratified by methods for evaluating frailty (mFI vs others). OS: Overall survival; RCC: Renal cell carcinoma.

**Figure 4.**
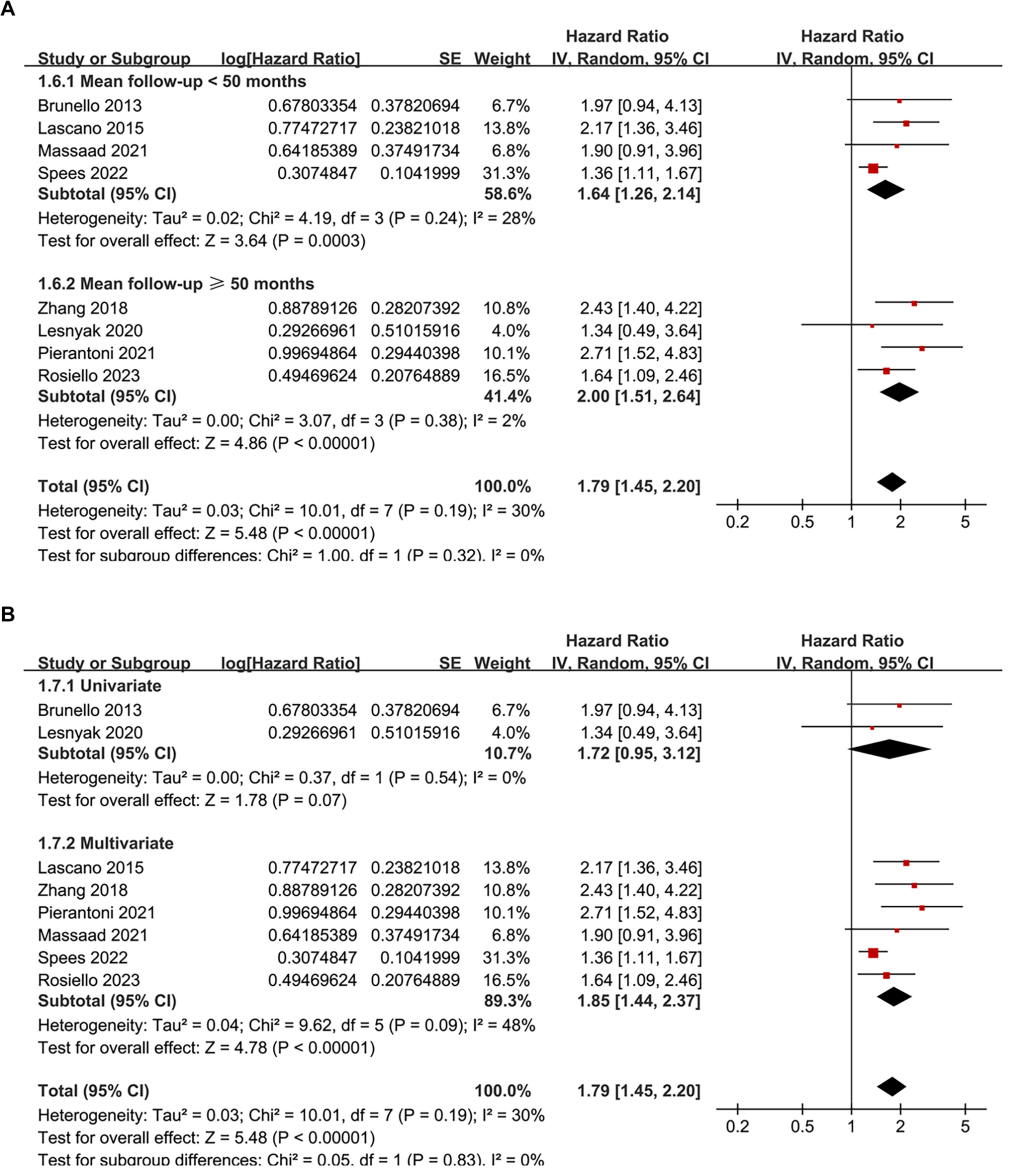
**Subgroup analyses of the association between frailty and OS of patients with RCC.** (A) Stratified by follow-up duration (< 50 vs ≥ 50 months); (B) Stratified by analytic models (univariate vs multivariate). OS: Overall survival; RCC: Renal cell carcinoma.

**Figure 5.**
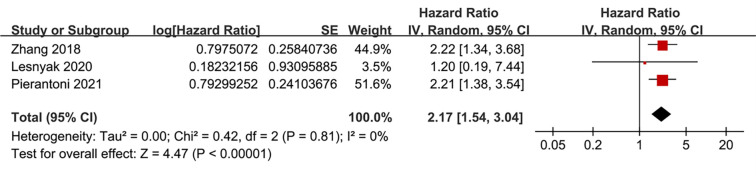
**Forest plot of the association between frailty and PFS of patients with RCC.** Pooled analysis comparing patients with and without frailty shows that frailty is significantly associated with poor PFS. PFS: Progression-free survival; RCC: Renal cell carcinoma.

Pooled analysis of eight studies [24–31] showed that frailty was associated with poor OS of patients with RCC (HR ═ 1.79, 95% CI: 1.45–2.20; *P* < 0.001; Figure 2A), with moderate heterogeneity observed across studies (*I*^2^ ═ 30%). To evaluate the robustness of the pooled results, a sensitivity analysis was conducted by sequentially omitting each included study. The overall association between frailty and poor OS remained statistically significant across all iterations, with pooled HRs ranging from 1.65 to 2.02, all with *P* < 0.05. Notably, the sensitivity analysis limited to studies with good quality (NOS ≥ 7) [25, 26, 28–31] showed similar results (HR ═ 1.85, 95% CI: 1.44–2.37; *P* < 0.001; *I*^2^ ═ 48%). Subsequently, subgroup analysis by cancer stage showed consistent associations between frailty and poor OS in non-metastatic (stage I-III) and metastatic (stage IV) RCC (HR: 1.92 vs 1.78, *P* for subgroup difference ═ 0.74; Figure 2B). In addition, consistent results were obtained for patients who received non-surgical or surgical treatments (HR: 1.71 vs 1.96, *P* for subgroup difference ═ 0.54; Figure 2C), for patients with mean ages <65 or ≥65 years (HR: 2.20 vs 1.63, *P* for subgroup difference ═ 0.15; Figure 3A), in studies with frailty evaluated with mFI and other scales (HR: 1.96 vs 1.71, *P* for subgroup difference ═ 0.54; Figure 3B), in studies with follow-up duration < or ≥ 50 months (HR: 1.64 vs 2.00, *P* for subgroup difference ═ 0.32; Figure 4A), and in studies with univariate and multivariate analyses (HR: 1.72 vs 1.85, *P* for subgroup difference ═ 0.83; Figure 4B). Finally, pooled results from three studies [26, 27, 29] showed that frailty was also associated with poor PFS of patients with RCC (HR ═ 2.17, 95% CI: 1.54–3.04; *P* < 0.001; *I*^2^ ═ 0%; Figure 5). Sensitivity analysis by excluding one study at a time showed similar results (HR: 2.12–2.21, all *P* < 0.05).

### Publication bias

**Figure 6.**
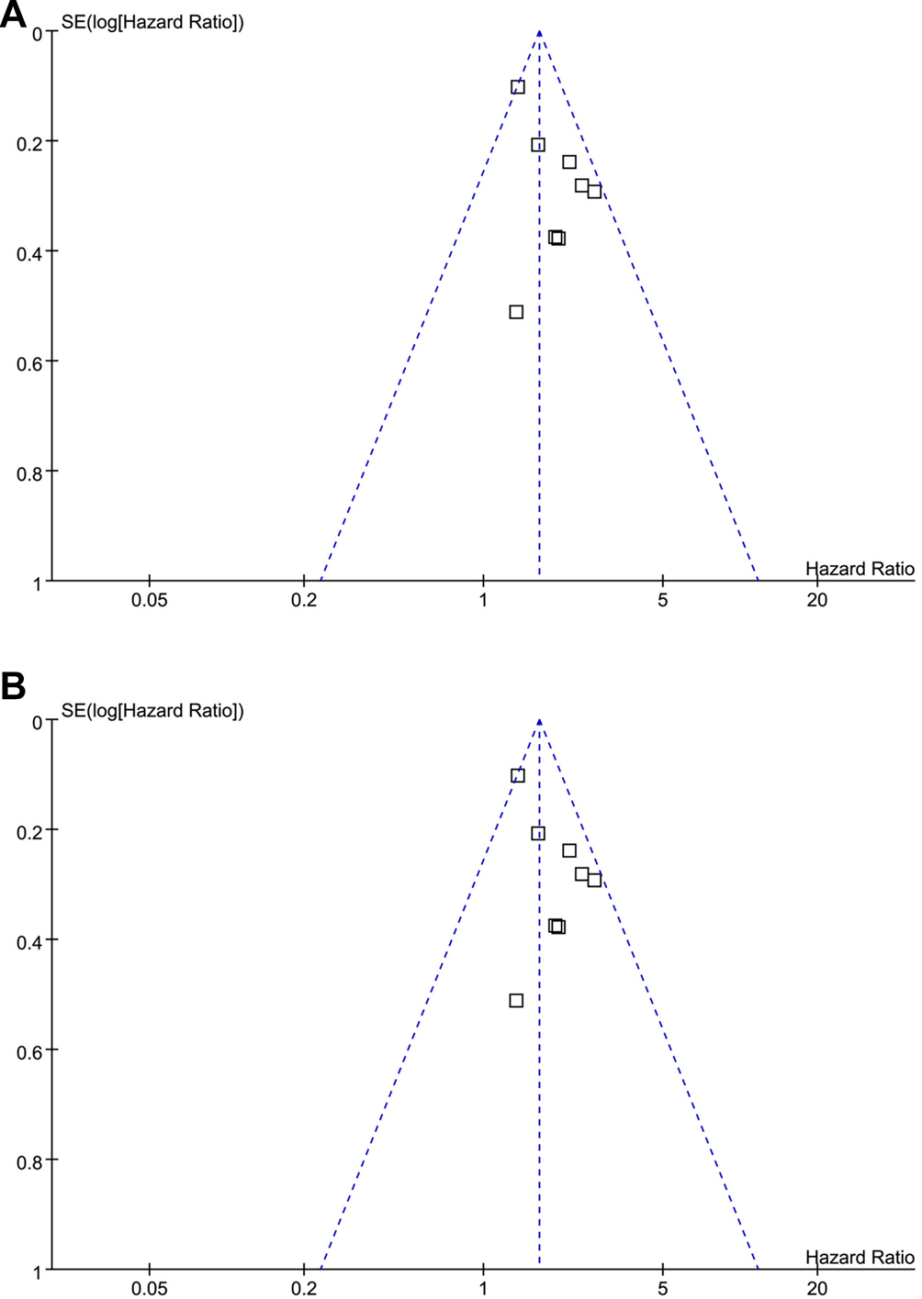
**Funnel plot assessing publication bias.** (A) Funnel plot for the meta-analysis of OS; (B) Funnel plot for the meta-analysis of PFS. OS: Overall survival; PFS: Progression-free survival.

The funnel plots assessing the association between frailty and OS/PFS of patients with RCC are presented in Figure 6A and 6B. Visual inspection of the plots suggests a symmetrical distribution, indicating a low likelihood of publication bias. For the meta-analysis of OS, this observation is further supported by Egger’s regression test, which yielded a non-significant result (*P* ═ 0.34). For the meta-analysis of PFS, Egger’s regression test was not performed because only three studies were included.

## Discussion

This meta-analysis of eight cohort studies involving 15,989 patients with RCC revealed a significant association between frailty and poor survival outcomes. Frailty was linked to a higher hazard of death and disease progression compared to non-frail patients. These associations were consistent across sensitivity analyses and various subgroups defined by cancer stage, treatment type, patient age, frailty assessment method, follow-up duration, and analytic model. Moderate heterogeneity was observed for OS, while no heterogeneity was found for PFS. These findings suggest that frailty may be a clinically meaningful prognostic indicator in RCC across diverse clinical settings.

The observed link between frailty and adverse survival outcomes in RCC is biologically and clinically plausible. Frailty is associated with chronic systemic inflammation, immune dysfunction, sarcopenia, and impaired physiological reserve, all of which can negatively influence a patient’s ability to respond to and recover from cancer treatment [32]. Inflammation-related markers, such as interleukin-6 and C-reactive protein, which are often elevated in frail individuals [33], are also known to promote tumor progression and metastasis [34]. In the context of RCC, a disease that can be highly angiogenic and immune-responsive [35], the presence of frailty may hinder the effectiveness of systemic therapies, such as tyrosine kinase inhibitors or immune checkpoint inhibitors, and increase susceptibility to treatment-related complications [36, 37]. Clinically, frailty may lead to treatment de-escalation, dose reductions, or delayed interventions, which could further compromise oncologic outcomes [38]. Furthermore, frailty often coexists with comorbidities and polypharmacy, increasing the risk of postoperative complications and limiting therapeutic options [39].

Subgroup analyses provided additional insights into the robustness and generalizability of our findings. The association between frailty and poor OS persisted across both non-metastatic (stage I–III) and metastatic (stage IV) RCC, suggesting that frailty exerts a negative prognostic impact independent of cancer stage. Similarly, frailty was predictive of worse OS in patients undergoing both surgical and non-surgical treatments, underscoring its relevance across different therapeutic strategies. The survival disadvantage associated with frailty was observed in both younger (<65 years) and older (≥65 years) populations, although the hazard appeared slightly greater among the younger group. This may reflect a more pronounced deviation from physiological baseline in younger frail patients or a more aggressive disease course in frailty-compromised individuals who would otherwise be expected to tolerate treatment. Subgroup analysis by frailty assessment methods also demonstrated consistent findings across different instruments (e.g., mFI vs others), suggesting the prognostic utility of frailty irrespective of the specific tool used.

This meta-analysis has several strengths. It is, to our knowledge, the first to comprehensively quantify the impact of frailty on survival outcomes in RCC, integrating data from diverse clinical settings and applying rigorous methodological standards in accordance with PRISMA guidelines. The included studies collectively represent a broad range of patient demographics, cancer stages, treatment modalities, and healthcare systems, enhancing the generalizability of the findings. Furthermore, the consistency of the results across multiple subgroup and sensitivity analyses lends confidence to the overall conclusions. Nonetheless, several limitations must be acknowledged. First, the majority of the included studies were retrospective in design, which may introduce selection bias and limit the ability to control for confounding factors [40]. Second, there was variability in the tools used to assess frailty, as well as in the definitions and cutoffs applied within each instrument. While this reflects real-world clinical heterogeneity, it may also affect the precision of pooled estimates. Third, the included studies differed in terms of treatment strategies, ranging from nephrectomy to targeted therapy, and such variation may influence the relationship between frailty and survival. Fourth, although most studies adjusted for key demographic and clinical covariates in multivariate models, residual confounding by unmeasured factors—such as performance status, nutritional status, and socioeconomic variables—cannot be ruled out. Fifth, causality cannot be inferred due to the observational nature of the included studies. Moreover, none of the included studies stratified outcomes by RCC histologic subtypes (e.g., clear cell, papillary, chromophobe), precluding analysis of potential subtype-specific differences in the prognostic value of frailty. Additionally, one study did not report the sex distribution of its participants [24]. However, as our meta-analysis pooled HRs based on the overall patient population rather than sex-specific estimates, the impact of this omission on the overall findings is expected to be minimal. Finally, PFS data were limited to only three studies, and the findings for this outcome should be interpreted with caution until further evidence becomes available.

From a clinical perspective, the findings of this meta-analysis support the routine integration of frailty assessment into the pre-treatment evaluation of patients with RCC. Identifying frail individuals at baseline could inform risk stratification, guide treatment planning, and prompt the implementation of supportive measures such as prehabilitation, nutritional optimization, and multidisciplinary care. In addition, standardized frailty screening could help clinicians individualize therapeutic decisions, balancing oncologic benefits against potential harms in vulnerable patients. Future research should focus on prospective studies to validate frailty as a prognostic marker in RCC, explore its interaction with specific treatment modalities, and assess the impact of frailty-targeted interventions on clinical outcomes. Harmonization of frailty assessment tools and development of RCC-specific frailty models may also enhance predictive accuracy and clinical utility.

In terms of implementation, specific frailty instruments may be selected based on clinical setting and resource availability. The mFI, with a threshold score of ≥ 0.27 (≥ 3 of 11 items), is suitable for high-throughput clinics, requiring only a brief review of electronic medical records and minimal staff time [41]. The Clinical Frailty Scale (CFS), where a score ≥ 5 indicates frailty, can be rapidly applied by trained clinicians or nurses through visual and functional judgment during outpatient visits [42]. The CGA [43], although more time- and resource-intensive (30–60 min by a multidisciplinary team), is ideal for pre-operative evaluations in older or complex patients and allows tailored interventions. A suggested workflow may involve initial screening with the CFS or mFI, followed by full CGA in patients flagged as frail. Adopting such structured approaches may facilitate routine frailty assessment in RCC care and enable more individualized decision-making.

## Conclusion

In conclusion, this meta-analysis indicates that frailty is significantly associated with worse survival outcomes in patients with RCC, underscoring its potential value in prognostication and treatment planning. Future prospective studies are needed to determine the optimal scale for frailty evaluation in patients with RCC, validate the association between frailty and poor prognosis, clarify the mechanisms linking frailty to cancer outcomes, and determine whether targeted interventions to address frailty can improve prognosis in RCC. Integrating standardized frailty screening into clinical pathways may help refine risk stratification and support more personalized approaches to RCC management.

## Supplemental data

**Detailed search strategy for each database**

*PubMed*

(“Frailty”[MeSH] OR frailty[tiab] OR frail[tiab]) AND (“Kidney Neoplasms”[MeSH] OR renal[tiab] OR kidney[tiab]) AND (cancer[tiab] OR tumor[tiab] OR carcinoma[tiab] OR neoplasm[tiab] OR adenoma[tiab] OR malignancy[tiab]) AND (“Recurrence”[MeSH] OR “Mortality”[MeSH] OR “Survival”[MeSH] OR “Prognosis”[MeSH] OR recurrence[tiab] OR death[tiab] OR mortality[tiab] OR survival[tiab] OR prognosis[tiab] OR deaths[tiab] OR remission[tiab] OR collapse[tiab] OR follow-up[tiab] OR followed[tiab] OR metastasis[tiab] OR progression[tiab] OR longitudinal[tiab] OR cohort[tiab] OR died[tiab])

*Embase*

(‘frailty’/exp OR frailty:ti,ab OR frail:ti,ab) AND (‘kidney tumor’/exp OR renal:ti,ab OR kidney:ti,ab) AND (cancer:ti,ab OR tumor:ti,ab OR carcinoma:ti,ab OR neoplasm:ti,ab OR adenoma:ti,ab OR malignancy:ti,ab) AND (‘recurrence’/exp OR ‘mortality’/exp OR ‘survival’/exp OR ‘prognosis’/exp OR recurrence:ti,ab OR death:ti,ab OR mortality:ti,ab OR survival:ti,ab OR prognosis:ti,ab OR deaths:ti,ab OR remission:ti,ab OR collapse:ti,ab OR follow-up:ti,ab OR followed:ti,ab OR metastasis:ti,ab OR progression:ti,ab OR longitudinal:ti,ab OR cohort:ti,ab OR died:ti,ab)

*Web of Science*

TS ═ (frailty OR frail) AND TS ═ (renal OR kidney) AND TS ═ (cancer OR tumor OR carcinoma OR neoplasm OR adenoma OR malignancy) AND TS ═ (recurrence OR death OR mortality OR survival OR prognosis OR deaths OR remission OR collapse OR follow-up OR followed OR metastasis OR progression OR longitudinal OR cohort OR died)

## Data Availability

All data generated or analyzed during this study are included in this published article.
